# *Trypanosoma cruzi* parasitemia in chronic Chagas disease: Insights from hierarchical modeling

**DOI:** 10.1371/journal.pntd.0010612

**Published:** 2022-08-04

**Authors:** Fernando Abad-Franch

**Affiliations:** Núcleo de Medicina Tropical, Faculdade de Medicina, Universidade de Brasília, Brasília, Brazil; Instituto de Ciências Biológicas, Universidade Federal de Minas Gerais, BRAZIL

## Parasitemia in theory: Frequency and intensity

I have recently suggested [1] that multilevel hierarchical models [2] can help us dissect, and hence understand, the complex process of detecting pathogens in their hosts. Although the problem of imperfect pathogen detection is pervasive, I focused on *Trypanosoma cruzi*, the agent of Chagas disease [3], as a motivating example [1]. In particular, [1] outlines a situation in which a group of patients with untreated, chronic Chagas disease (CCD) is studied by drawing replicate blood samples from each patient and then testing each sample with replicate tests aimed at detecting *T*. *cruzi* [1]. The “detection-history” data thus generated (a series of detections/nondetections from the replicate tests) can then be used to estimate, under a set of assumptions [1,2], 2 critical parameters governing the process of *T*. *cruzi* detection: (i) the probability that the target parasite is present, and hence available for detection, in a blood sample drawn from an infected patient—i.e., sample-level target availability, denoted θ; and (ii) the probability that the target is detected by a test that is run on a sample where the target is available—i.e., “true” test sensitivity, denoted *p*. If enough information is available, one may also estimate the effects of covariates on those 2 probabilities [1,2].

Within this general framework, θ can be seen as a measure of how often, on average, parasites circulate in the bloodstream of CCD patients. Estimates of θ thus provide insight into the frequency at which the transient “pulses” of parasitemia typical of CCD [3–6] may be expected to occur. Since parasites can effectively only be “picked” in either blood samples or vector blood meals during those pulses, knowledge of θ can critically advance our understanding of both parasite-based diagnosis and *T*. *cruzi* transmission dynamics. For any given test, moreover, *p* measures the probability that at least one parasite is detected in a sample that contains the target parasites [1]. Importantly, this probability will increase, all else being equal, as parasite density increases [7]. It follows that variations in test-specific *p* estimates can provide insight into the relative amount of parasites that circulate in the patient’s bloodstream during pulses of parasitemia—all else being equal, smaller versus larger values of *p* should signal lower- versus higher-intensity parasitemias [7].

This line of reasoning, therefore, suggests that estimates of θ and *p*, if available, would provide insight into, respectively, (i) the frequency of *T*. *cruzi* parasitemia pulses and; (ii) the intensity of that parasitemia in CCD patients. I believe that such knowledge could substantially sharpen our view of within-host parasite dynamics, likely with major implications for understanding *T*. *cruzi* transmission and CCD epidemiology, diagnosis, or treatment. However, formal estimates of θ and *p* are just lacking for *T*. *cruzi*—and indeed for most human pathogens [1]. Why? For one thing, the fact that pathogen detection is a naturally hierarchical problem [1] does not seem to be widely appreciated. As a consequence, most practitioners either run single tests, often on single samples, or aggregate replicated results in summary or composite measures; the former strategy does not yield the information needed to formally separate θ from *p*, and the latter loses or disregards that information [1].

In this report, I use publicly available, real-life data to illustrate how the hierarchical-modeling approach described in [1] can be used in practice to estimate θ and *p* and to investigate whether and how those parameters vary, e.g., over time or with patient characteristics. Because in this case, the data were available before I developed my hypotheses, I first describe the data and then lay out my hypotheses.

## Parasitemia in practice 1: The data

The data are from 91 untreated CCD patients described in [5]. Overall, 23 of them had indeterminate CCD, 23 had mild heart disease (stages C1 to C3), and 45 had more severe heart disease (stages C4 or C5; see [5,8]). All patients were first tested by using 5-mL aliquots of a 35-mL venous blood sample for (i) a 6-tube blood culture plus (ii) a duplicate qPCR assay; 44 of the 91 patients were similarly retested 2 to 3 years later using a second blood sample [5]. Patient age (mean ± SD, 51.9 ± 11.2 y; range, 25 to 81) was recorded at the time of drawing the first blood sample. The patient- and sample-specific number of blood culture tubes that were positive for *T*. *cruzi* is available in Tables 1 and 2 of [5]. However, qPCR results were reported as either the “mean ± SD parasite load” (in parasite equivalents mL^−1^) or “negative”; because the SD of parasite load was >0 for all positive samples, here I will assume that the duplicate qPCR assays run on each of those samples both yielded detections—and that neither did in negative samples. The raw data used as input for the present analyses are available in S1 Dataset. I note that in this particular dataset, there is some evidence (e.g., from Kruskal–Wallis and Tukey tests) that indeterminate-phase patients were overall younger than those with symptomatic disease; I therefore did not fit any models including both patient condition and patient age as covariates on either θ or *p*; rather, I asked which of the 2 correlated variables better helps explain variation in those parameters [9].

## Parasitemia in practice 2: The hypotheses

The null hypothesis states that both (i) the frequency of *T*. *cruzi* parasitemia pulses (measured by θ); and (ii) the intensity of parasitemia (approximated by *p*) were constant over time and across patient traits. A “completely null hypothesis” also states that sensitivity was the same for qPCR and blood culture. Because this is extremely unlikely, I also considered a “realistic null hypothesis” in which *p* was allowed to vary between tests—in the expectation that sensitivity estimates would be much larger for qPCR than for blood culture [1,3,5,6,10].

Hypotheses about the frequency of parasitemia pulses refer to variation in θ. Specific versions state (i) that θ changed between the first and the second samples; (ii) that parasitemia pulses were more frequent (so that θ is larger) in more severely diseased patients; or, alternatively, (iii) that θ changed with patient age.

Hypotheses about parasitemia intensity refer to whether patient traits could help explain variation in *p* beyond that explained by between-test differences in sensitivity. Specific versions explored the possibilities that (i) parasitemia was more intense (so that *p* was larger) in more severely diseased patients or, alternatively, (ii) *p* changed with patient age (see below).

## Parasitemia in practice 3: General formulation of the models

To get a sense of the relative support that each hypothesis finds in the data, I built a set of 3-level hierarchical models [2,11] representing specific hypotheses about θ and *p* (as outlined above) plus some “combined” models with covariate effects on both θ and *p* (Table 1). Because all patients were infected [5], infection probability (denoted Ψ) was fixed at 1.0 in all models. Models were fitted by maximum likelihood in the free software Presence 2.13.4 [12]. These models accommodate missing test result data by simply skipping them in the computation of the likelihood [1,2,11]; this was the case of all second-sample results for the 47 patients who were not retested [5]. Three-level models also account for the nonindependence of results arising from repeated sample testing [2]. Model performance was compared using sample size-corrected (*N* = 91 patients) Akaike’s information criterion (AICc) scores and Akaike weights (*w*_i_) [9]. Better-performing models have smaller AICc scores and larger weights; they represent a better compromise between model fit (as evaluated by the likelihood) and parsimony (as measured by the number of estimable parameters) than do competing models with larger AICc scores and smaller *w*_i_ values [9]. Since each model can be mapped to a specific hypothesis about how the system works, differences in AICc and *w*_i_ gauge the relative support that each of those competing hypotheses finds in the data [9]. The 3-level hierarchical models used here [2] can in general be represented by “M_Ψ/θ/*p*_ = Ψ(…); θ(…); *p*(…)”, with level-specific covariates written inside the parentheses after Ψ, θ, and *p* (Table 1). I give some examples below.

**Table 1:** The complete 20-model set.

Model*	AICc	ΔAICc	*w*_i_	*k*	Deviance
Ψ(1.0); θ(age); *p*(test+condition) [“M_Top_”]	1,030.61	0	0.3227	6	1,015.26
Ψ(1.0); θ(sample+age); *p*(test+condition)	1,031.11	0.50	0.2514	7	1,013.35
Ψ(1.0); θ(.); *p*(test+condition)	1,031.47	0.86	0.2099	5	1,018.47
Ψ(1.0); θ(sample); *p*(test+condition)	1,031.96	1.35	0.1643	6	1,016.61
Ψ(1.0); θ(condition); *p*(test+condition)	1,036.07	5.46	0.0210	7	1,018.31
Ψ(1.0); θ(sample+condition); *p*(test+condition)	1,036.69	6.08	0.0154	8	1,016.47
Ψ(1.0); θ(age); *p*(test)	1,039.70	9.09	0.0034	4	1,028.99
Ψ(1.0); θ(sample+age); *p*(test)	1,040.07	9.46	0.0028	5	1,027.07
Ψ(1.0); θ(.); *p*(test) [“Realistic null”]	1,040.55	9.94	0.0022	3	1,032.08
Ψ(1.0); θ(sample); *p*(test)	1,040.93	10.32	0.0019	4	1,030.22
Ψ(1.0); θ(age); *p*(test+age)	1,041.53	10.92	0.0014	5	1,028.53
Ψ(1.0); θ(sample+age); *p*(test+age)	1,041.97	11.36	0.0011	6	1,026.62
Ψ(1.0); θ(.); *p*(test+age)	1,042.30	11.69	0.0009	4	1,031.59
Ψ(1.0); θ(sample); *p*(test+age)	1,042.73	12.12	0.0008	5	1,029.73
Ψ(1.0); θ(condition); *p*(test)	1,044.94	14.33	0.0002	5	1,031.94
Ψ(1.0); θ(sample+condition); *p*(test)	1,045.45	14.84	0.0002	6	1,030.10
Ψ(1.0); θ(condition); *p*(test+age)	1,046.80	16.19	0.0001	6	1,031.45
Ψ(1.0); θ(sample+condition); *p*(test+age)	1,047.37	16.76	0.0001	7	1,029.61
Ψ(1.0); θ(.); *p*(.) [“Completely null”]	1,203.85	173.24	0	2	1,197.57
Ψ(1.0); θ(1.0); *p*(test+condition) [θ fixed at 1.0]	1,220.24	189.63	0	5	1,207.24

*The models have 3 hierarchical levels: (i) host-level infection, Ψ, which here was fixed at 1.0 because all patients were known to be infected [5]; (ii) sample-level parasite availability, θ; and (iii) test-level sensitivity, *p*. Covariates in θ and/or *p* submodels (type of **test**, first/second blood **sample**, patient **age**, and patient clinical **condition**) are indicated in parentheses, with a dot representing a submodel without covariates. Models are ranked by sample size-corrected AICc scores; ΔAICc is, for any given model, the difference between its AICc and the AICc of the top-ranking model (here, “[M_Top_]”); *w*_i_ are Akaike weights, which can be interpreted as the weight of evidence for each model and its associated hypothesis, relative to the set of models (and hypotheses) under consideration; *k* is the number of estimable parameters (a measure of model complexity); and the deviance is twice the negative log-likelihood of each model (a measure of model fit).

AICc, Akaike’s information criterion corrected for small sample size.

## Insight 1: Null hypotheses

The “completely null” model, “M_1/null/null_ = Ψ(1.0); θ(.); *p*(.)”, estimates mean θ at 70.3% (95% confidence interval (CI) 61.9% to 77.5%) and mean *p* at 44.1% (40.5% to 47.8%); it has an AICc score of 1,203.85 (Table 1). Model “M_1/null/test_ = Ψ(1.0); θ(.); *p*(test)” represents the “realistic null hypothesis” that bloodstream parasite availability was constant (θ = 69.8%, CI 61.5% to 77.0%) but blood culture and qPCR had different sensitivities: 31.3% (CI, 27.6% to 35.3%) for each blood culture tube and 83.8% (77.8% to 88.5%) for each qPCR assay. The AICc score of this model is 1,040.55, which is 163.30 units smaller than that of the “completely null” model (Table 1); this is overwhelming evidence [9] against “M_1/null/null_” and, therefore, against the “completely null hypothesis”. Null-model analyses, in sum, (i) show that, as expected, θ was <100%; and (ii) provide very strong support to the view that (also as expected) qPCR was, on average, much more sensitive than blood culture at detecting *T*. *cruzi* in blood samples that contained the target. It seems therefore unreasonable to model the data without specifying test differences in sensitivity, and all models below include test effects on *p*.

## Insight 2: Bloodstream parasite availability

Model “M_1/sample/test_ = Ψ(1.0); θ(sample); *p*(test)” allows θ to vary between the first and the second blood samples, which were drawn 2 to 3 y apart [5]. This model has an AICc score of 1,040.93—i.e., 0.38 units larger than that of “M_1/null/test_” (Table 1). This indicates that the evidence that θ varied between the 2 samples is at best faint; the numerical output of “M_1/sample/test_” suggests that parasitemia might have been, if anything, somewhat more common at the second (77.5%, CI 62.8% to 87.5%) than at the first sampling (66.1%, CI 55.8% to 75.1%). The widely overlapping CIs and the fact that the AICc score of “M_1/sample/test_” is larger than that of the more parsimonious “M_1/null/test_” both cast doubt on this sample effect—for which, moreover, there would seem to be little biological justification given that patients did not take anti-*T*. *cruzi* drugs between the first and the second sampling [5]. These results thus suggest that, as expected, between-sample differences in parasite availability were all but negligible.

Is there any evidence that θ varied with patient traits? Model “M_1/condition/test_ = Ψ(1.0); θ(condition); *p*(test)” represents the hypothesis that pulses of parasitemia may vary in frequency depending on whether patients had indeterminate, mild, or severe CCD (see [5] and above). This model clearly underperforms “M_1/null/test_” (AICc 4.39 units larger) and estimates near-zero effects of clinical condition on θ, providing compelling evidence against the associated hypothesis—bloodstream parasite availability did not seem to change much among patients with indeterminate (71.6%, CI 54.7% to 84.1%), mild (70.8%, 53.6% to 83.6%), or severe CCD (68.4%, 56.2% to 78.5%). (Note, in addition, how the wider CIs for the first 2 conditions appropriately reflect the larger uncertainty brought about by the smaller size of those 2 groups—23 patients each [5].)

How about patient age? One might hypothesize, for example, that bloodstream parasite pulses might increase in frequency as patients get older and their immune system senesces. Alternatively, smaller θ values might be more common among older patients if more frequent pulses of parasitemia (larger θ) were associated with earlier mortality. The first hypothesis predicts a positive effect of patient age on θ, whereas the second predicts a negative age effect. These hypotheses can both be represented by model “M_1/age/test_”; the sign of the age coefficient estimate (β_age-θ_) will suggest which one has more support from the data. The AICc of this age-θ model (1,039.70) is smaller than that of “M_1/null/test_” (1,040.55), suggesting some age effects on θ; as predicted by the second version of the age-θ hypothesis, the slope coefficient estimate for age is negative (β_age-θ_ = −0.0307, SE 0.0128), suggesting that older CCD patients tended to have less frequent parasitemia pulses. Under this model, θ values are expected to range from 49.3% (CI, 30.2% to 68.6%) for the oldest (81 y) to 84.4% (71.2% to 92.2%) for the youngest (25 y) patients in the sample—with θ = 70.3% (61.9% to 77.5%) for mean-age patients (52 y). Models with test-specific *p* and a more complex structure on θ had larger AICc scores than “M_1/age/test_” (Table 1).

Overall, then, these parasite-availability analyses suggest that pulses of *T*. *cruzi* parasitemia were (i) about as frequent when the first blood sample was drawn as they were 2 to 3 years later; (ii) about as frequent in indeterminate-phase patients as they were in those with heart disease, whether mild or severe; and (iii) somewhat rarer in older patients—which raises the intriguing (and worth investigating) possibility of a negative association between θ and life expectancy in CCD patients. I again stress that parasite-availability estimates were around 70% (from approximately 50% to approximately 85%), not 100%; a model with θ fixed at 1.0 (“M_1/1/test_”) had an AICc score >180 units larger than those of models (including “M_1/null/test_”) in which θ was estimated from the data (Table 1). Hence, the idea that *T*. *cruzi* was always available for detection in any blood sample drawn from these chronically infected patients had no empirical support whatsoever.

## Insight 3: Bloodstream parasite density

The results from the “realistic null model” described above show that, when parasites were available for detection in a blood sample, sensitivity was much higher for qPCR than for blood culture. Considering that, for a given test, target detection probabilities should correlate with target abundance [7], I further suggested that we can gain insight into parasitemia intensity by evaluating whether and how test-specific sensitivities vary with patient traits.

For example, median parasite load (as reported in [5]) was nearly twice as high in mildly diseased patients (0.44 parasite equivalents mL^−1^) as in those with severe heart disease (0.23), and was much lower in indeterminate-disease patients (0.09 parasite equivalents mL^−1^). This suggests that, when parasitemia is present, the amount of circulating parasites may vary depending on clinical condition—and one may hypothesize that this should affect the average sensitivity of both blood culture and qPCR [7]. Model “M_1/null/test+condition_ = Ψ(1.0); θ(.); *p*(test+condition)” represents this hypothesis. This model clearly outperforms all previous specifications, with an AICc score 9.08 units smaller than that of “M_1/null/test_”; by itself, this result suggests that sensitivity was not only different for each test—it also varied among patients with different disease conditions. Model “M_1/null/test+condition_” estimates:

- Bloodstream parasite availability at 69.9% (CI, 61.6% to 77.1%);
- Single-tube blood culture sensitivity at 23.4% (17.7% to 30.3%) for indeterminate-disease patients, at 41.7% (34.3% to 49.5%) for mildly diseased patients, and at 30.1% (25.1% to 35.6%) for severely diseased patients; and
- Single qPCR assay sensitivity for the same clinical condition groups at, respectively, 78.4% (69.3% to 85.4%), 89.5% (83.8% to 93.3%), and 83.6% (76.8% to 88.7%).

These sensitivity estimates closely parallel median parasite-load values—lowest in indeterminate-phase patients, highest in mild chronic disease, and intermediate in severe chronic disease [5]. Although based on just 3 data points, there are very strong positive correlations between qPCR-measured median parasite loads [5] and test-sensitivity estimates—with R^2^ values consistently above 0.99. The upper half of S1 Fig shows those correlations for *p* estimates derived from model “M_1/null/test+condition_”.

To see if there was any evidence that the density of parasites in each pulse of parasitemia changed with patient age, I tested for common effects of age on *p* for both blood culture and qPCR. Model “M_1/null/test+age_”, which represents this hypothesis, did not have any support from the data—its AICc score was 10.83 units larger than that of “M_1/null/test+condition_”, and the age coefficient estimate was effectively indistinguishable from zero (β_age-*p*_ = −0.0054, SE 0.0061).

Taken together, these results suggest that, during pulses of parasitemia, *T*. *cruzi* bloodstream populations were probably somewhat denser (more parasites per unit blood volume) in mildly diseased than in severely diseased patients—and somewhat less dense in indeterminate-phase patients. In contrast, bloodstream parasite density was overall age independent.

## Insight 4: The full model set—Relative support, effects, and predictions

I fitted a set of “combined” models to investigate possible effects of sample (first versus second) and patient characteristics (age or clinical condition) on θ and *p* (Table 1). The top-performing (smallest AICc) model in the full, 20-model set was of the form “M_Top_ = M_1/age/test+condition_”; it estimates a negative effect of age on θ (β_age-θ_ = −0.0314, SE 0.0128) and positive effects of mild (β_mild-*p*_ = 0.8544, SE 0.2367) and severe clinical condition (β_severe-*p*_ = 0.3478, SE 0.2121) on test sensitivity (*p*). The second-ranking model included age and sample as covariates on θ (Table 1), but θ estimates were similar for the first (θ_1_ intercept = 2.3494, SE 0.7162) and the second (θ_2_ intercept = 2.9340, SE 0.7911) samples. Two further models were within 2 AICc units of the top-ranking model (Table 1); one had no covariates on θ, and the other estimated θ at 66.2% (CI, 55.9% to 75.2%) for the first sample and 77.6% (CI, 62.9% to 87.6%) for the second. Other competing models had little to no empirical support, with ΔAICc values ≥5.46 and Akaike weights *w*_i_ ≤ 0.021 (Table 1).

**Fig 1:**
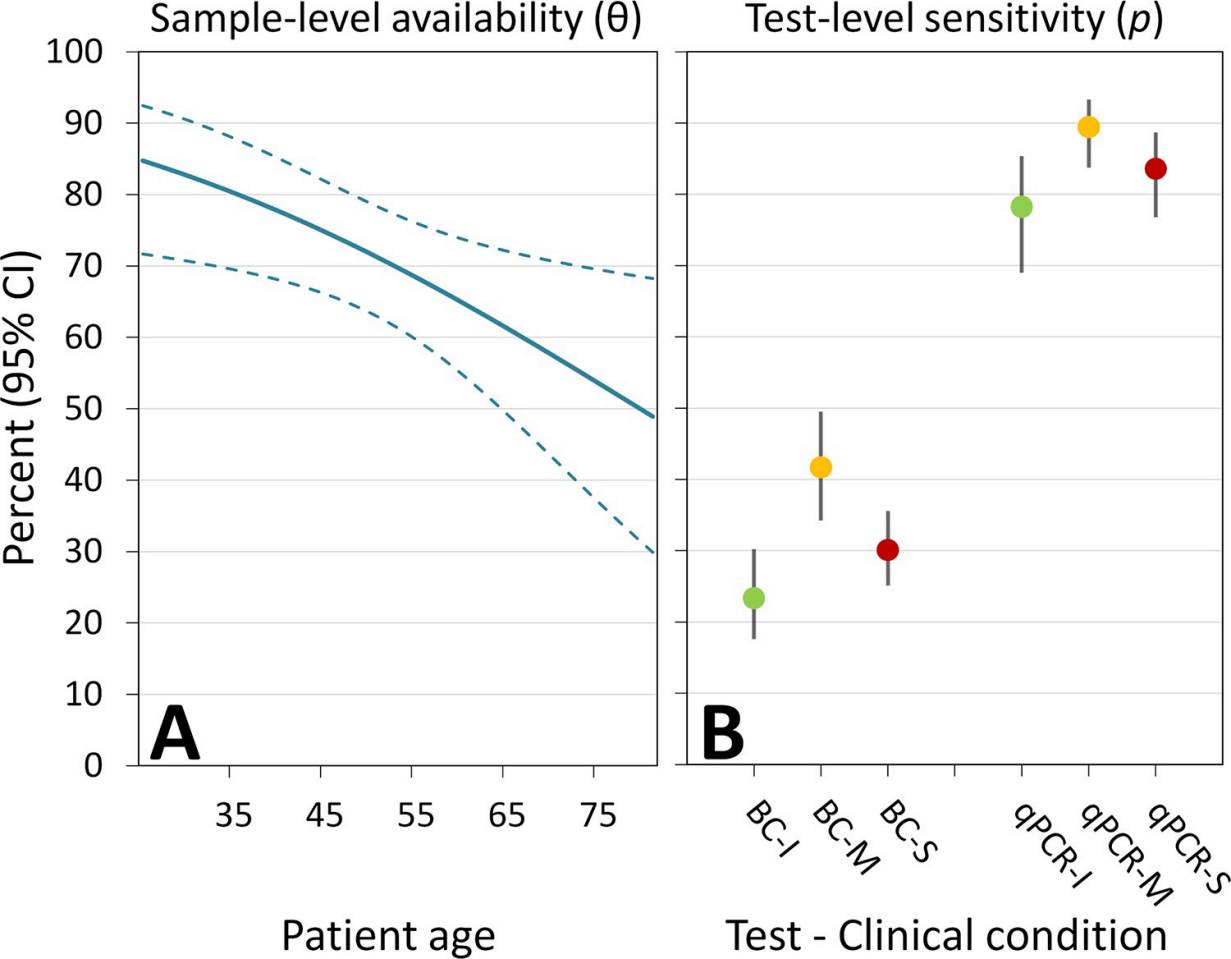
Availability of *T*. *cruzi* in blood samples drawn from chronically infected patients (θ, panel A) and sensitivity of diagnostic tests run on samples in which *T*. *cruzi* was available for detection (*p*, panel B). The percentages and 95% CIs are predictions from the top-ranking model (“M_Top_ = M_1/age/test+condition_”) in a set of 20 competing hierarchical models (see Table 1). In panel (**A**), patient age is shown in years; in panel (**B**), the results refer to 2 types of tests (blood culture, “BC”; and “qPCR”) and 3 patient conditions: indeterminate (“I”), mild (“M”), and severe (“S”) chronic Chagas disease. Sample-level availability (θ) can be seen as a measure of the frequency at which parasitemia “pulses” occur, and differences in test-specific sensitivities (*p*) across clinical conditions as an approximation to the intensity of those pulses of parasitemia—all else being equal, denser bloodstream parasite populations will lead to increased sensitivities (see [7] and S1 Fig). Numerical values used to build these graphs are provided in S1 Table.

Fig 1 shows the top-ranking model estimates of bloodstream parasite availability (θ) and test sensitivities (*p*) and how they varied, respectively, with patient age (between 25 and 81 y) and with diagnostic test (1 blood culture tube or 1 qPCR assay) and patient clinical condition (indeterminate, mild, or severe CCD [5]). Together with the insights outlined above, these results suggest (i) that *T*. *cruzi* bloodstream forms were typically present in approximately 70% (not 100%) of venous blood samples; (ii) that the frequency of those pulses of parasitemia did not vary much either between 2 samples drawn 2 to 3 y apart or among patients in different clinical conditions, but decreased moderately with patient age (Fig 1A); and (iii) that, in patients experiencing a pulse of parasitemia, the intensity of that parasitemia was lowest in indeterminate-phase patients, intermediate in severely diseased patients, and highest in mildly diseased patients—with little, if any, variation with patient age (Fig 1B). Again, test sensitivity estimates were very strongly (R^2^ > 0.99), positively correlated with condition-specific median parasite loads as reported in [5] (see lower half of S1 Fig).

## Caveats and conclusions

Here I have illustrated, using real-life data, how a hierarchical-modeling approach can provide crucial insight into the dynamics and detection of *T*. *cruzi* bloodstream forms in CCD patients. It is my view that estimates like the ones discussed here, and especially those presented in Fig 1, are far more informative than measures of bloodstream parasite availability that disregard imperfect (<100%) test sensitivity or measures of test sensitivity that disregard imperfect (<100%) parasite availability [1]. The former are often presented as estimates of the “infectiousness” of pathogen-carrying hosts to blood-feeding vectors (e.g., [13]), and the latter as estimates of the “clinical sensitivity” of diagnostic procedures (cf. [14]); as a rule, a formal assessment of how test sensitivity affects measures of “infectiousness”, and of how pathogen availability affects measures of “clinical sensitivity”, is lacking in these kinds of studies.

I finally emphasize that the caveats and model assumptions (about, e.g., tests being approximately 100% specific or individuals being independent with respect to infection status) discussed more thoroughly in [1,2] also apply to the results presented here—which, importantly, are intended to illustrate the approach but do not provide clinical guidance. I note, moreover, that whether the present results generalize beyond the 91 patients in [5] is unclear and should be investigated. For example, anti-*T*. *cruzi* treatment [15,16], parasite lineage [17], or patient traits not considered here (sex, comorbidities, nutritional or immune status…) may all be hypothesized, along with other factors, to affect the dynamics of parasitemia. Likewise, the sensitivity of particular test types may vary across laboratories or with more specific factors such as equipment, protocols, or test-user skills (see, e.g., [10]). With data from “robust designs” (i.e., replicate testing of replicate samples [1,2,7,11,18]), the approach outlined here provides a rigorous means to investigate these and similar hypotheses.

Keeping these caveats in mind (see [1,2]), my analyses suggest, in sum, that the pulses of *T*. *cruzi* parasitemia typical of CCD [3–6] were probably more frequent in younger patients (Fig 1A), and that the density of bloodstream parasites associated with those pulses probably varied with disease severity (Fig 1B). Neither parasite availability (θ) nor test sensitivity (*p*) was 100%; instead, θ estimates ranged from approximately 50% to approximately 85%, depending on patient age, and *p* estimates ranged from approximately 20% to approximately 90%, depending on diagnostic test and patient clinical condition (Fig 1 and S1 Table). These findings are overall in line with the hypothetical scenario described in [1], although they incorporate some of the complexity one would expect to come across in real-life settings. At any rate, they provide a sharp illustration of how hierarchical modeling can help us develop a stronger understanding of pathogen population dynamics and diagnosis—with potentially important implications for both clinical practice and epidemiology.

## Supporting information

S1 Dataset The raw data used in the analyses. For each sample/test combination, “1” denotes detection, “0” denotes nondetection, and “-” denotes that the test was not run.
(XLSX)

S1 Fig Correlations between test sensitivity estimates and median parasite loads. Sensitivity estimates are derived from 2 of the models fitted here (“M_1/null/test+condition_”, upper half; and “M_Top_”, lower half; see Table 1 of the main text), and parasite loads are as reported in [5].
(TIF)

S1 Table Numerical values used to build Fig 1 of the main text. For Fig 1A, predicted values of sample-level target availability (θ) and 95% CIs across patient ages (from 25 to 81 y); for Fig 1B, predicted values of test-specific sensitivities (*p*) and 95% CIs across patient clinical conditions.
(XLSX)
