# Supplementary figures and images for: Trypanosoma cruzi parasitemia in chronic Chagas disease: Insights from hierarchical modeling
Source: PLoS Negl Trop Dis. 2022 Aug 4;16(8):e0010612. doi: 10.1371/journal.pntd.0010612 (PMC9352048; doi:10.1371/journal.pntd.0010612)

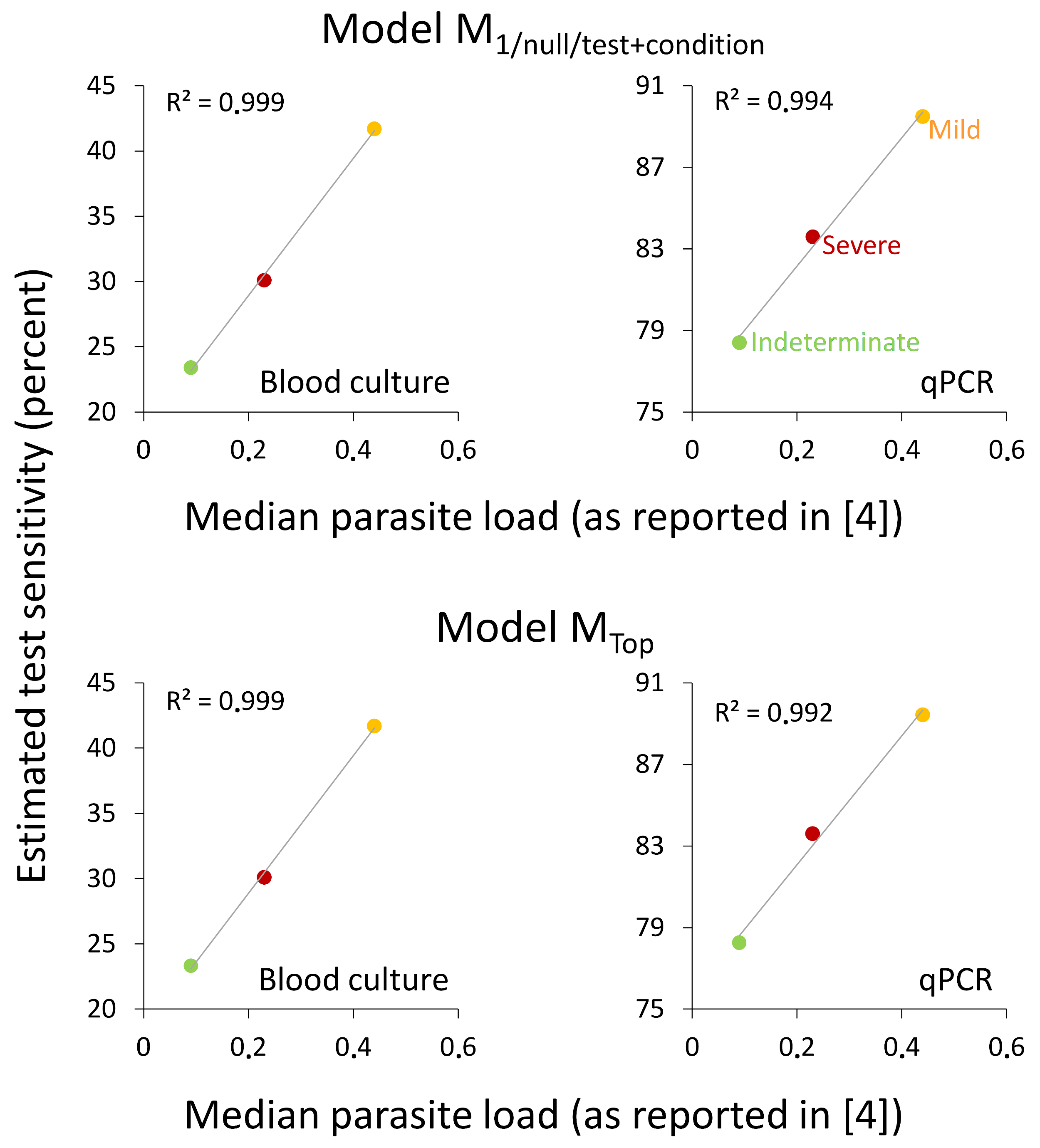

Supplement: S1 Fig — Sensitivity estimates are derived from 2 of the models fitted here (“M1/null/test+condition”, upper half; and “MTop”, lower half; see Table 1 of the main text), and parasite loads are as reported in [5]. (TIF) [file pntd.0010612.s002.tif]
